# An evaluation of meniscal collagenous structure using optical projection tomography

**DOI:** 10.1186/1471-2342-13-21

**Published:** 2013-07-23

**Authors:** Stephen HJ Andrews, Janet L Ronsky, Jerome B Rattner, Nigel G Shrive, Heather A Jamniczky

**Affiliations:** 1 Department of Biomedical Engineering, Schulich School of Engineering, University of Calgary, Human Performance Lab, 2500 University Drive NW, Calgary, AB T2N 1N4, Canada; 2 Mechanical and Manufacturing Engineering, Schulich School of Engineering, Faculty of Kinesiology, University of Calgary, Calgary, Canada; 3 McCaig Institute for Bone and Joint Health, University of Calgary, Calgary, Canada; 4 Department of Cell Biology and Anatomy, The McCaig Institute for Bone and Joint Health, Faculty of Medicine, University of Calgary, Calgary, Canada

**Keywords:** Meniscus, Optical projection tomography, Collagen structure

## Abstract

**Background:**

The collagenous structure of menisci is a complex network of circumferentially oriented fascicles and interwoven radially oriented tie-fibres. To date, examination of this micro- architecture has been limited to two-dimensional imaging techniques. The purpose of this study was to evaluate the ability of the three-dimensional imaging technique; optical projection tomography (OPT), to visualize the collagenous structure of the meniscus. If successful, this technique would be the first to visualize the macroscopic orientation of collagen fascicles in 3-D in the meniscus and could further refine load bearing mechanisms in the tissue. OPT is an imaging technique capable of imaging samples on the meso-scale (1-10 mm) at a micro-scale resolution. The technique, similar to computed tomography, takes two-dimensional images of objects from incremental angles around the object and reconstructs them using a back projection algorithm to determine three-dimensional structure.

**Methods:**

Bovine meniscal samples were imaged from four locations (outer main body, femoral surface, tibial surface and inner main body) to determine the variation in collagen orientation throughout the tissue. Bovine stifles (n = 2) were obtained from a local abattoir and the menisci carefully dissected. Menisci were fixed in methanol and subsequently cut using a custom cutting jig (n = 4 samples per meniscus). Samples were then mounted in agarose, dehydrated in methanol and subsequently cleared using benzyl alcohol benzyl benzoate (BABB) and imaged using OPT.

**Results:**

Results indicate circumferential, radial and oblique collagenous orientations at the contact surfaces and in the inner third of the main body of the meniscus. Imaging identified fascicles ranging from 80-420 μm in diameter. Transition zones where fascicles were found to have a woven or braided appearance were also identified. The outer-third of the main body was composed of fascicles oriented predominantly in the circumferential direction. Blood vessels were also visualized using this technique, as their elastin content fluoresces more brightly than collagen at the 425 nm wavelength used by the OPT scanner.

**Conclusions:**

OPT was capable of imaging the collagenous structure, as well as blood vessels in the bovine meniscus. Collagenous structure variability, including transition zones between structural regions not previously described in the meniscus, was identified using this novel technique.

## Background

The menisci are complex three-dimensional structures with a heterogeneous structure. The semi-lunar, wedge shapes of the menisci increase the contact area between the rounded femoral condyles and the relatively flat tibia plateau, thereby protecting the articular cartilage from excessive stresses [1]–[3]. Menisci are fibrocartilages, which have been described as having intermediate structural and functional properties between those of dense, fibrous connective tissue (i.e. ligament, tendon) and hyaline cartilage [4].

The composition and structure of the meniscus has been studied extensively since Fairbank discovered the relationship between meniscal removal and the observation of degenerative changes in the knee [5]. An early investigation of the menisci by Bullough et al. in [6], described the structure of human menisci as consisting predominantly of circumferentially oriented fibres [6]. The study also identified the presence of radially oriented tie-fibres that the authors supposed acted to tie the circumferentially oriented fibres together to prevent longitudinal splitting of the tissue. The conceptual model of a predominance of circumferentially oriented fibres has subsequently been supported in numerous studies [7]–[11].

The major limitation of these previous models is the use of two-dimensional (2D) imaging techniques to evaluate a highly three-dimensional (3D) structure. One can section and image 2D structures sequentially and subsequently reconstruct 3D models, but this technique is time consuming and difficult, and to date has not been completed for the meniscus. Optical sectioning using confocal microscopy can yield 3D structures, but the maximum sample depth of a few hundred microns is a limitation in this technique.

Optical projection tomography (OPT) is a promising technique which could overcome the inherent difficulties in imaging a highly heterogeneous structure [12], as it is an imaging technique capable of imaging samples on the meso-scale (1-10 mm) at a micro-scale resolution. The technique takes two-dimensional images of objects from multiple angles and reconstructs them using a back projection algorithm to determine its three-dimensional structure). It is optically similar to computed tomography (CT), but uses white or fluorescent light rather than x-ray to image the object of interest. The technique requires for the tissue to be cleared to allow light to pass through the object. OPT has primarily been used to evaluate development and gene expression in mouse embryos [12]. To date, no study has attempted to evaluate collagenous structure of connective tissues using OPT. As collagen and elastin both autofluoresce at similar wavelengths (325 nm) [13] in the fluorescent spectrum, this technique may be useful in the evaluation of meniscal tissue structure. The study described here was exploratory in nature. The purpose was to determine the ability of OPT to visualize the collagenous matrix organization of the meniscus.

## Methods

### Sample preparation

**Figure 1:**
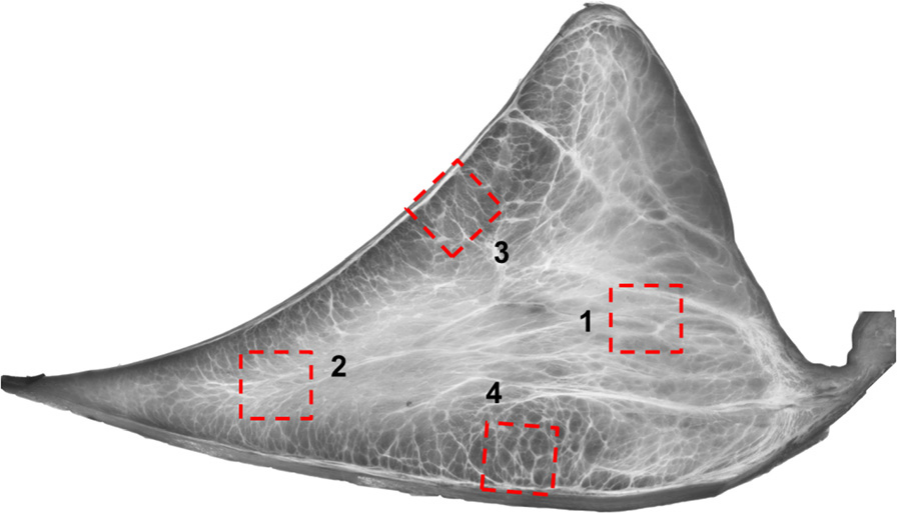
**Illustration of sample locations obtained for OPT. 1**) Outer-third main body **2**) Inner third main body **3**) Femoral surface **4**) Tibial Surface.

Bovine stifle joints (n = 2) were obtained from a local abattoir within 48 hours of slaughter (18-30 months age) and the medial menisci were carefully dissected. The menisci were fixed in 100% methanol at 4°C for 48 hours. Four specimen (approximately 3-4 mm in each of three dimensions) were dissected from a radial section cut from the anterior portion of the meniscus and prepared for OPT (Figure 1). The scanner is capable of imaging samples up to 10 mm. However, as the scanner resolution is inversely related to the sample size, sample dimensions of 3-4 mm were chosen. The four specimens were prepared from different locations in order to observe site-specific variation in the structure. The specimens were prepared from: 1) Outer third of the main body 2) Inner third of the main body 3) Femoral surface 4) Tibial Surface. Institutional review board approval was not required for this study.

### Optical projection tomography

Specimens were scanned using fluorescence optical projection tomography [12] on a Bioptonics 3001 M OPT scanner (Bioptonics Microscopy, Edinburgh). Each tissue sample was embedded in a block of 1.5% low melting point agarose (Invitrogen). Blocks were trimmed and glued to mounts, and dehydrated through three washes of 100% methanol (Fisher) over 24 hours. Specimens were then cleared for 24 hours in BABB (1 part benzyl alcohol (Fisher): 2 parts benzyl benzoate (Sigma) in a fume hood, allowing any remaining methanol to evaporate.

Native autofluorescence was imaged using the GFP-1 channel (exciter 425 nm/40 nm; emitter LP475 nm) at a resolution of 13.6-20 μm. Raw images were reconstructed into greyscale slices using NRecon (Skyscan NV, Kontich). Each reconstruction results in a stack of 250-500 images, based on the sample size and resolution used. Three dimensional reconstructions were then carried out using ImageJ (NIH open source software) [14]. The stacks of images were imported and visualized using the 3D Viewer plugin and the Volume Viewer plugin. The 3D Viewer renders a 3D surface image of the sample from the slices while the volume viewer allows visualization of sections through the sample in a user defined plane. Images of interest were exported to Adobe Photoshop Elements 9.0 (Adobe Inc.) and filtered with a despeckling filter. Measurements of fascicle diameter were completed using ImageJ pixel information and converting to length using the known resolution of the images.

## Results

**Figure 2:**
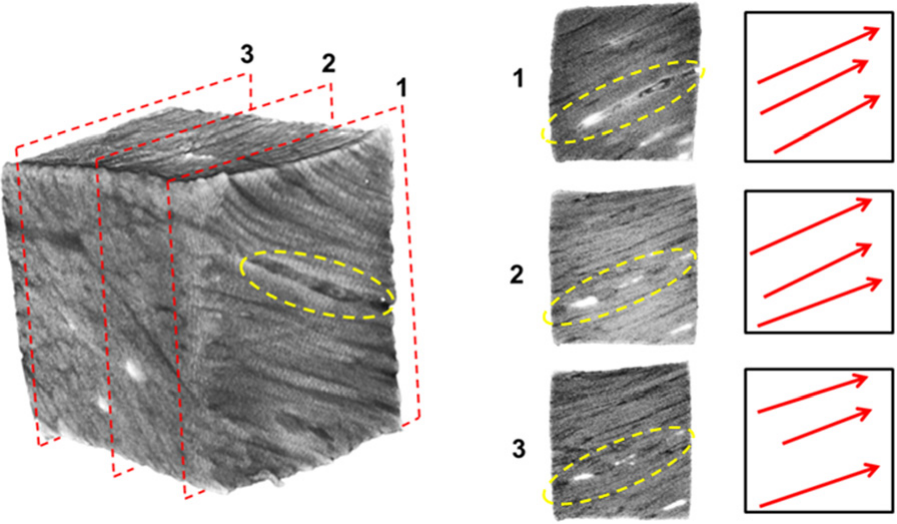
**A meniscal sample taken from the outer one-third of the main body of the meniscus.** The planes identified by the red dashed lines are shown in the panels **1-3** to the right. Predominant fascicle directions are illustrated by the red arrows. All planes showed similar fascicle orientations. The collagen sparse void space containing blood vessels are indicated by the yellow dashed ellipses.

**Figure 3:**
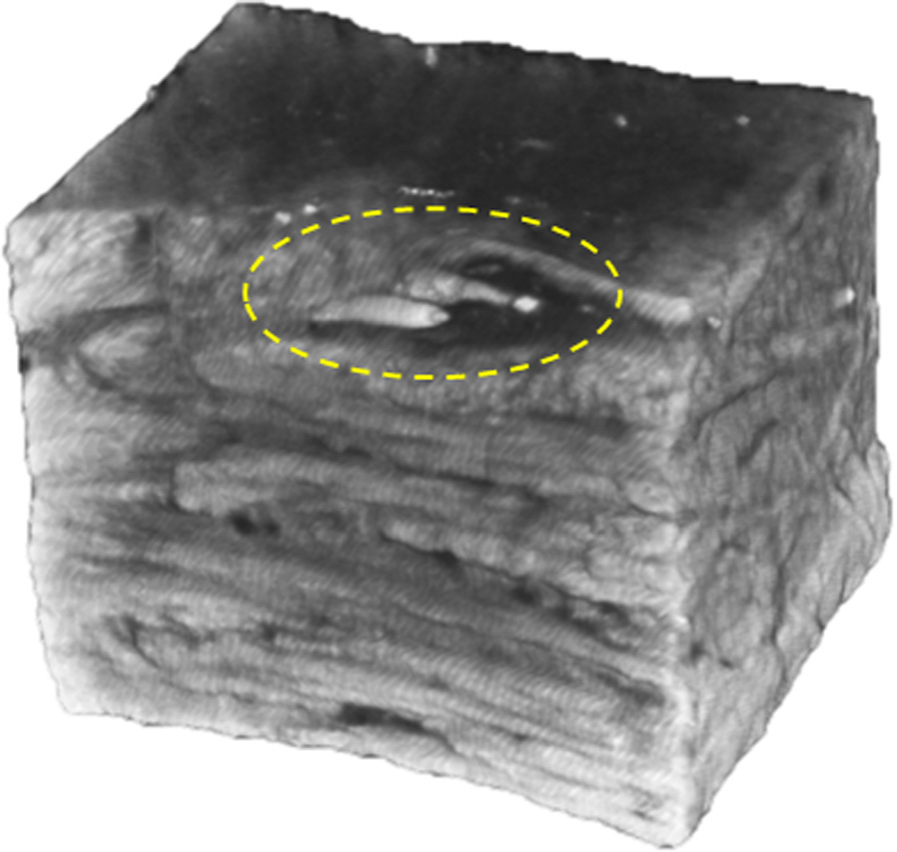
**A meniscal specimen taken from the femoral surface of a medial meniscus.** Two blood vessels can be seen running parallel inside an area devoid of collagen fascicles.

**Figure 4:**
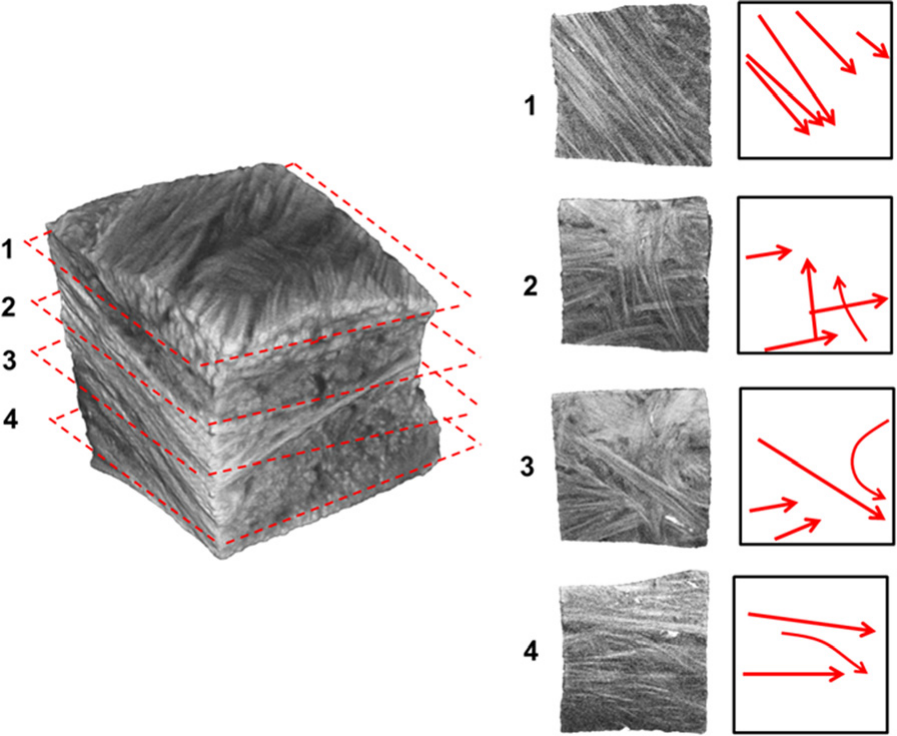
**A meniscal sample taken from the inner one-third of the main body of the meniscus.** Varying fascicle orientations can be observed in planes **1-4**, moving in the superior to inferior direction. Red arrows to the right indicate the predominant fascicle directions in each breakout section image.

**Figure 5:**
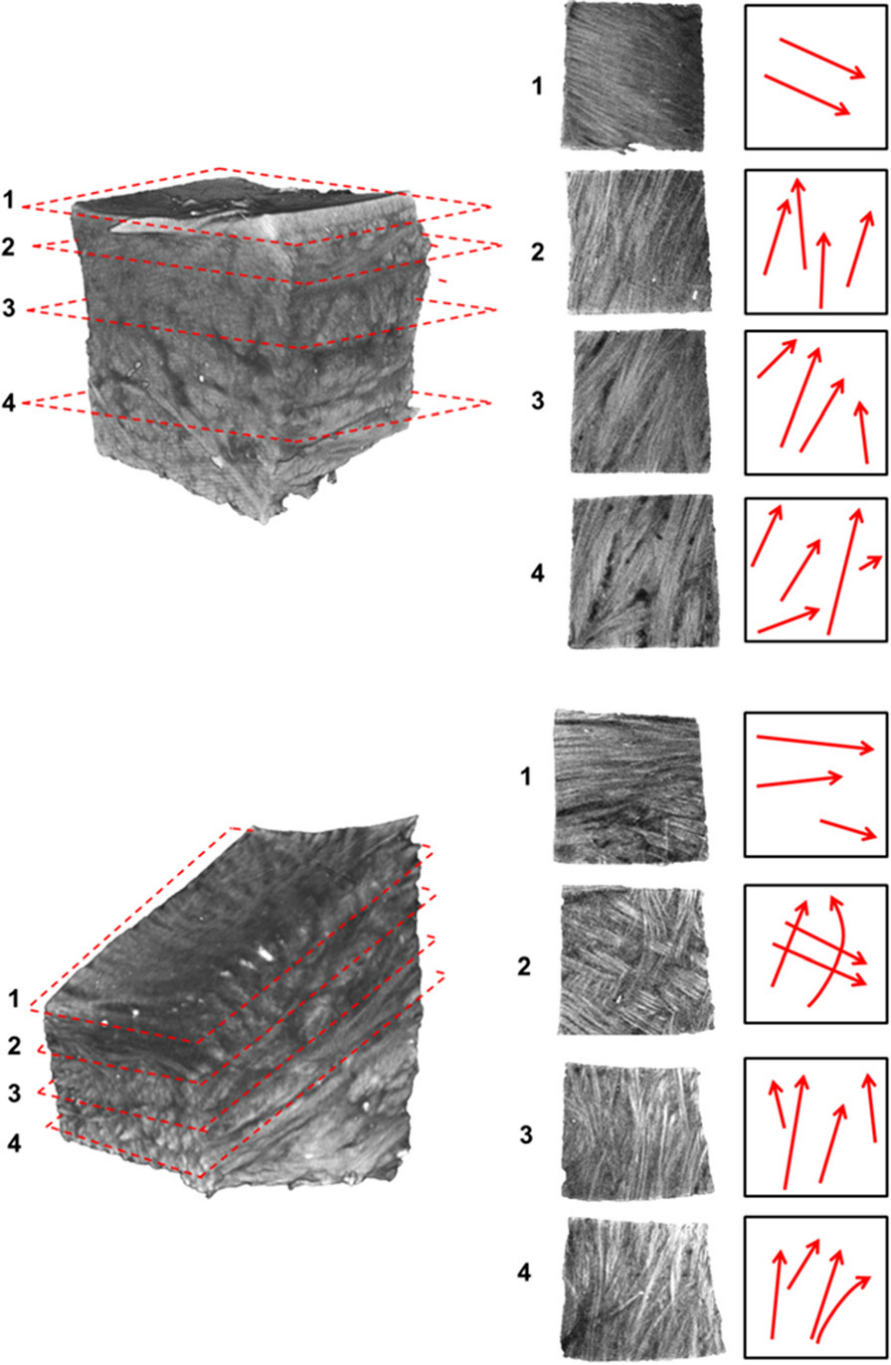
**Meniscal specimens dissected from the femoral surface (top) and tibial surface (bottom) of a medial meniscus.** Fibre bundles at the lamellar layer are oriented in the radial direction parallel to the meniscal surface (**1**). Moving through the tissue, in the direction normal to the surfaces, the fascicle directions transition to braided organizations: sections **2-3** (top), sections **3-4** (bottom) in a direction oblique to the lamellar layer. Woven fascicle organization can be seen in both specimen; section 4 (top) and section 2 (bottom).

**Figure 6:**
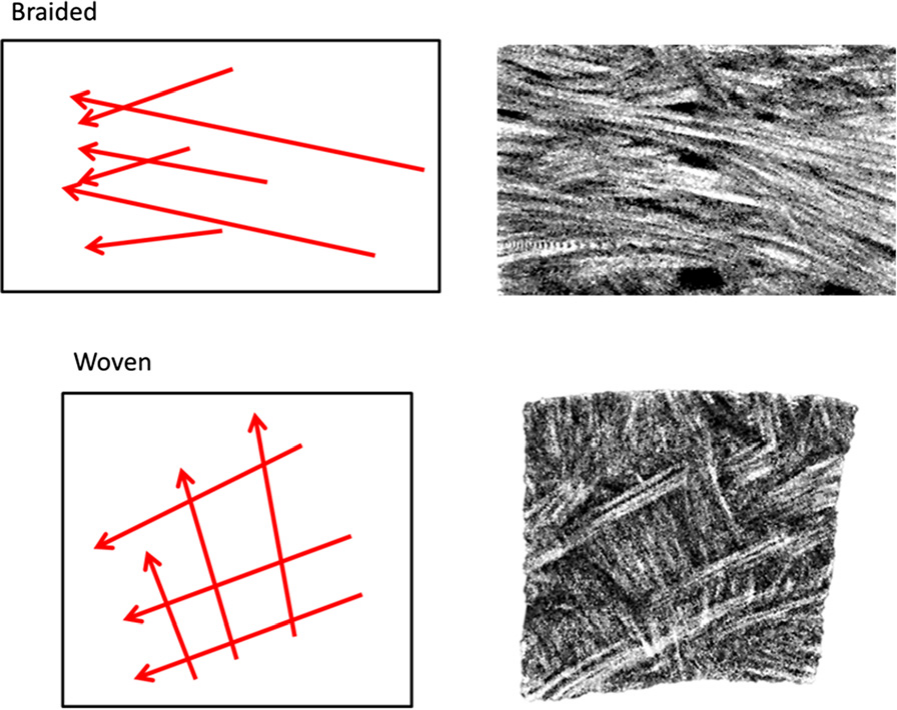
**Schematic representation (left) of braided and woven fascicle organizations with associated sections from meniscal samples illustrating these arrangements (right).** Scale bars 1 mm.

Optical projection tomography was capable of imaging collagen fascicle orientations in 3D within a specimen. We were also able to identify blood vessels in all specimens obtained from the outer half of bovine menisci. Specimens imaged with OPT each showed different fascicle orientation patterns. Specimen 1, taken from the outer third of the main body, contained a highly aligned fascicle structure. Sections through the middle, and the extreme ends of the specimen all showed the same predominant fascicle direction (Figure 2). Blood vessels, which fluoresce more brightly than the surrounding collagen, were seen to persist throughout the specimen. Multiple vessels appear to be situated in an area of low collagen density (Figure 2) Blood vessels were observed in samples taken from both the surfaces and outer portion of the menisci (Figure 3). An additional movie file shows this in more detail (see Additional file 1). Specimen 2 was taken in the same orientation as specimen 1, but from the inner third of the tissue. This specimen showed a distinctly different fascicle orientation pattern than specimen 1. The top surface of this sample was composed of a woven pattern with fascicles oriented perpendicular to others within the same plane (Figure 4). Moving through this section in the superior/inferior direction revealed high variability in the fascicle direction. The most superior plane contained aligned fascicles. Moving through the section revealed an intricately woven arrangement with fascicles oriented in multiple directions. The most inferior section through this specimen showed an aligned arrangement, oblique to the arrangement seen in the superior portion of the specimen. The samples from the femoral and tibial surfaces (specimen 3 and 4) demonstrated similar fascicle orientation patterns throughout (Figure 5). The contact surfaces contained fascicles oriented in the direction parallel to the surface in the radial direction. Moving in the direction normal to the contact surfaces, the fascicles changed alignment and in some sections became woven, and in others appeared to be braided. The braided appearance is defined by fascicles running obliquely to one another, rather than perpendicular as in the woven arrangement (Figure 6). Surface samples also revealed fluorescence indicative of blood vessels located beneath the lamellar layer. The fascicle size ranged from approximately 80-420 μm. These accuracy of these measurements are limited by manual detection of fascicle borders as well as the resolution of the pixels (14-20 μm based on the sample size).

## Discussion

This study has evaluated the ability of optical projection tomography to visualize the collagenous fascicle structure of meniscal specimen. The technique was able to resolve meniscal collagen fascicle orientation, differences between structural layers and blood vessels in three-dimensional space. Specimens dissected from the tibial and femoral surfaces demonstrated a highly aligned lamellar layer near the contact surfaces. This finding agrees with previous findings that showed a distinct layer (150-200 μm thick) with fascicles oriented in the radial direction along the surface of human menisci [10].

The maximum resolution of the system is approximately 5 μm. The resolution of the images obtained here ranged from 13.6-20 μm. Due to the limitation of this resolution, the imaging of individual collagen fibres (5 μm diameter [11]) is not possible, but collagen fascicles which have a diameters of 80-420 μm could be visualized. These fascicle sizes agree very well with previous scanning electron microscopy studies which found fascicle sizes on the range of 100-400 μm [10]. Further, visualizing the surface layer identified by Peterson and Tillman (10 μm thick) is not practical with samples of this size. This layer may be visible in smaller samples where the system resolution can be maximized.

The specimen from the outer third of the main body demonstrated the aligned circumferential fibre direction described previously [6,9,11]. This specimen also contained multiple blood vessels which were situated within a collagen-sparse void space. Recent unpublished work from our lab has indicated that this void space is likely a proteoglycan rich region which may play a protective role for blood vessels by shielding them from shear stresses. Blood vessels were also seen along the femoral and tibial surfaces. The position of the vasculature within the tissue agree with the findings of Arnoczky and Warren in the human meniscus [15].

We have identified two major types of fascicle organizations, braided and woven (Figure 6), not previously described in the meniscus. These two organizations could play a significant role in the load bearing mechanism and the fracture toughness of the meniscus. The braided fascicle organization is common in various types of rope [16]. This organization results in a non-linear stiffness which increases with increasing deformation. As the fascicles are stretched axially, they compress against adjacent fascicles increasing the friction between them, thereby increasing the stiffness. This is a mechanical behaviour also observed in meniscal samples in tension [17,18]. The woven arrangement is common in fabrics and in structures that withstand compressive loading onto a flat structure (e.g. a basket). This fascicle arrangement allows for the conversion of compressive forces into tensile forces in the weave via attachments to a rigid supporting structure. In the case of the menisci, the ligamentous attachments to the tibial plateau would provide a sufficient supporting structure. These two fascicle organizations could also play a role in increasing the fracture toughness of the menisci. A crack passing through a woven arrangement would encounter fascicles perpendicular to the direction of crack propagation, which would increase the size of deformation zone and divert energy away from the crack tip [19]. The bending of fascicles around one another would also increase the path length and direction a crack would have to follow in order to separate the fascicles. These combined mechanisms may increase the menisci’s resistance to failure under physiologic loading conditions. It was observed that many fascicles bifurcate or arborize into multiple smaller fascicles. These arborized fascicles tended to change direction from the path of the branching fascicles. Fascicles were often observed to curve within a plane (Figure 4: Image 3). These findings are also supported by recent findings from our lab [20]. These frequent changes in direction and arborization of fascicles make it difficult to quantify fascicle orientations as well as fascicle size distribution.

Tissue composition varies from the inner cartilage-like tip, to the outer edge of the meniscus [8,21,22]. The results of this study have demonstrated that the structure of the tissue is also transitional. From the outer third of the meniscus to the inner third the fascicle arrangement changes from being highly aligned in the circumferential direction to being a woven, less organized structure at the inner third. The inner tip is similar to hyaline cartilage in both composition and structure. Proteoglycan content is approximately 10% and type II collagen is more abundant than type I collagen [21,23,24]. Taken together, it can be observed that from the inner tip to the outer edge, the bovine meniscus transitions from a hyaline cartilage-like tissue through an intermediate structure in the inner-portion to a highly aligned ligament-like portion at the outer edge. Further, at the contact surfaces there is a transition from the radially oriented lamellar layer at the surface to the woven or braided orientations seen in the sub-lamellar main body. Benjamin and Ralphs previously described fibrocartilage as a transitional tissue, as it is commonly seen in the interface between different tissue types (i.e. at the tendinous junctions with bone) and because of changes in tissue structure throughout development [25]. It has been demonstrated here that the structure within the meniscus also fits the paradigm of fibrocartilage as a transitional tissue.

## Conclusion

OPT was able to visualize collagen fascicle organization in bovine menisci. This novel technique had not previously been used in the study of connective tissues. We have visualized two fascicle organizations, not previously described in the menisci: braided and woven which may help in further elucidating the load bearing mechanisms within this highly complex tissue. Optical projection tomography is a promising technique in the evaluation of the organization of other connective tissues and changes that occur due to injury or disease processes. For example, changes that occur to tendons after implantation as anterior cruciate ligament (ACL) autografts could be examined to understand remodeling that occurs in soft tissue. This technique also has potential in developmental studies, evaluating collagenous matrix organization of connective tissues throughout the process of growth and maturation.

## Abbreviations

BABB: Benzyl alcohol benzyl benzoate; GFP: Green fluorescent protein; OPT: Optical projection tomography.

## Competing interests

The authors declare that they have no competing interests.

## Authors’ contribution

SA was the primary author and responsible for data analysis for this article. JLR was involved in project conception and article revision. NS provided expertise in the mechanical interpretation of the results and article revision. JBR aided in image analysis and article preparation. HJ provided expertise in sample preparation and data collection for OPT as well and article revision and writing. All authors read and approved the final manuscript.

## Pre-publication history

The pre-publication history for this paper can be accessed here:

http://www.biomedcentral.com/1471-2342/13/21/prepub

## Supplementary Material

Additional file 1 **A 3-D image (from ImageJ) of a meniscal sample taken from the femoral surface of a bovine meniscus.** As the image is rotated, a large void space can be observed in the sample (t = 10 seconds). Upon further rotation, the image is then zoomed in on two blood vessels which reside in parallel in the aforementioned void space. Click here for file
